# The Estimated Prevalence of Autism in School-Aged Children Living in Rural Nepal Using a Population-Based Screening Tool

**DOI:** 10.1007/s10803-018-3610-1

**Published:** 2018-05-31

**Authors:** Michelle Heys, Felicity Gibbons, Ed Haworth, Emilie Medeiros, Kirti Man Tumbahangphe, Mary Wickenden, Merina Shrestha, Anthony Costello, Dharma Manandhar, Elizabeth Pellicano

**Affiliations:** 1 UCL Institute for Global Health, University College London, London, UK; 2 Great Ormond Street Institute of Child Global Health, University College London, London, UK; 3 Chelsea and Westminster NHS Foundation Trust, London, UK; 4 Mother and Infant Research Activities, Kathmandu, Nepal; 5 Autism Care Society, Kathmandu, Nepal; 6 Tribhuvan University Teaching Hospital, Department of Child Health, Kathmandu, Nepal; 7 Department of Maternal, Newborn, Child and Adolescent Health (MCA), World Health Organization, Geneva, Switzerland; 8 Centre for Research in Autism and Education (CRAE), UCL Institute of Education, University College London, London, UK; 9 Department of Educational Studies, Macquarie University, Sydney, Australia

**Keywords:** Autism, Prevalence, Screening

## Abstract

Few data exist on the prevalence of autism in low-income countries. We translated, adapted and tested the acceptability of a Nepali-language version of a screening tool for autism (Autism Quotient-10). Using this tool, we estimated autism prevalence in 4098 rural Nepali children aged 9–13 years. Fourteen children scored > 6 out of 10, indicative of elevated autistic symptomatology, of which 13 also screened positive for disability. If the AQ-10 screening tool is as sensitive and specific in the Nepali population as it is in the UK, this would yield an estimated true prevalence of 3 in 1000 (95% confidence interval 2–5 in 1000). Future research is required to validate this tool through in-depth assessments of high-scoring children.

**Electronic supplementary material:**

The online version of this article (10.1007/s10803-018-3610-1) contains supplementary material, which is available to authorized users.

## Introduction

Autism is a global phenomenon. An estimated 1–2% of children worldwide lie on the autism spectrum, with approximately 52 million autistic^1^ individuals across the globe (Baxter et al. 2015). These estimates are largely driven, however, by prevalence estimates from high-income countries (HIC). Virtually no data exist on the population prevalence of autism in low-income countries (LIC) and only one in a LIC rural setting (Uganda: Kakooza-Mwesige et al. 2014). In fact, there is a paucity of research examining autism generally in these regions at all (Abubakar et al. 2016). Studies that have been conducted in lower-middle and upper-middle income countries have produced varied results reporting prevalence estimates ranging from 0.32 (China: Tao 1987) to 250 per 10,000 (China: Ren et al. 2003) and more recently 90 per 10,000 (India: Raina et al. 2017). These discrepancies are possibly due to a variety of reasons, including the fact that autism is a spectrum condition (American Psychiatric Association 2013), the variety of traits; changing definitions of autism; varying levels of awareness in different countries; cultural variation in expectations and understandings of children’s behavior; different methodological approaches used to assess prevalence; and the lack of availability of culturally-sensitive diagnostic tools and year of assessment (Elsabbagh et al. 2012).

Variation in methodological approaches to assessing autism prevalence includes differences in case-finding techniques, from population-based sampling (Baird et al. 2006) to sampling from clinics and healthcare registers (Croen et al. 2002). Variation is also found in the method of diagnosing or screening cases for autism, including a mix of relying on healthcare or educational reports (Idring et al. 2012) and/or researchers assessing for autism first-hand using comprehensive diagnostic tools including the Autism Diagnostic Observation Schedule (ADOS) and the Autism Diagnostic Interview (ADI) (Kim et al. 2011), to less sensitive screening tools or questionnaires, based on different standards and diagnostic criteria (Stewart and Lee 2017). Furthermore, while many studies have investigated the prevalence of autism in Western societies, the general consensus is that there is an urgent need for more studies investigating the prevalence of autism in LICs (Abubakar et al. 2016; Elsabbagh et al. 2012; Mpaka et al. 2016; Ruparelia et al. 2016), in particular LIC rural settings using low-cost, community-based screening tools that can be administered by lower cadres of health workers. Without these tools and studies, it is impossible to draw an accurate prediction of the global prevalence of autism and develop appropriate services that can cater for the needs of autistic children and adults and their families.

Sixty-one percent of the world’s population of children and young people live in LICs or lower- to middle-income countries (LMIC). Nepal is one of the poorest LICs (World Bank 2016) and one of only four LICs outside of sub-Saharan Africa. The majority (estimated 82%) of its population lives rurally (World Bank 2014). Nepal has a growing population of about 26.7 million people due to high birth rate and declining death rate. The Autism Care Nepal Society website states that there is “no reliable estimate for the prevalence of autism in Nepal as autism is not known to many people” (Autism Care Society Nepal). Indeed, recent qualitative evidence from this population shows limited understanding of many aspects of atypical child development, in particular autism—but a strong desire to advocate for and increase support for these children and their families (Heys et al. 2016).

The overarching aim of the current study was to establish the prevalence of autism in school-aged children living in Nepal. In a preliminary screening-adaptation and acceptability study, we first sought to identify a low cost, short, population-based screening tool with good sensitivity and specificity that could potentially be delivered by all cadres of health care workers to detect possible autism in children and young people in a rural Nepali setting. We then sought to develop a Nepali-language adaptation of the identified screening tool, the AQ-10 (Allison et al. 2012), which would be acceptable for Nepali parents.

In the prevalence study, we used the resulting tool to estimate the prevalence of autism in 9–13 year-old Nepali children living in Makwanpur District, a rural hill area in the central region in which most households are dependent on subsistence agriculture (population > 500,000 in 2014). We also assessed the validity of the adapted AQ-10 within the same population by comparing results of a screening tool for childhood disability, which included questions on social and communication skills, and was delivered 6 months previously to the same cohort of children.

Our research aims were twofold: (1) to examine whether the AQ-10 is acceptable for use with Nepali parents and (2) to determine the estimated prevalence of autism in a rural population of Nepali children using the adapted AQ-10.

## Method

### Study Overview

Here, we describe two interrelated studies. The first study was designed to assess the adaptation and acceptability of a population-based screening tool. The second was a population-based prevalence study using the adapted screening tool. Data collection for the prevalence study occurred in two waves, wave 1 from Jan 2014 to July 2014 (during which a wide range of demographic and clinical data were obtained, including disability prevalence) and wave 2 from Sept 2014 to Jan 2015 (during which the autism screening was conducted). Description of sample size, inclusion and exclusion criteria and variables of interest are outlined in the two study sections.

### Preliminary Screening Tool Adaptation and Acceptability Study

There were three stages to this adaptation study: (1) the identification of the best fit screening tool, (2) translation and back translation of the chosen tool, and (3) a qualitative study on its acceptability.

#### Identification of Screening Tool

To begin, we conducted a review of available literature on population-based screening tools for autism using the search terms, ‘autis*’, ‘ASD’ and ‘screening’ in PubMed and the reference lists of existing articles. The key characteristics of these tools, including length of administration, cost, population target, evidence base, specificity and sensitivity and reporter characteristics (e.g., parent versus clinician report), were identified. Our criteria for tool selection for the current study included: (1) being population based, (2) for use across childhood and adolescence; (3) being freely available; (4) being short in length of administration; (5) requiring parent report only; and (6) having demonstrated sufficiently high sensitivity and specificity. Supplementary Table 1 shows the 12 screening tools that were identified, of which only one (the AQ-10; Allison et al. 2012) fitted best our criteria.

#### Adaptation and Translation of the Screening Tool

The Autism Quotient-10 (AQ-10; Allison et al. 2012) was identified in the review of screening tools as the most feasible for use in this population-based study, where the tool would be administered by trained Nepali field research assistants. It is a short version of the AQ-50, which was originally developed as a tool for screening for autistic traits in intellectually able adults (Baron-Cohen et al. 2001). A cut-off score of 6 or more out of 10 is used to identify children and young people who have a likely diagnosis of autism, with excellent sensitivity (93%) and specificity (97%) in UK populations (Allison et al. 2012). It has a child, adolescent and adult version, requires only limited specific knowledge of ASD, is administered in a short amount of time and is freely available.

Next, we translated (and back-translated) the AQ-10 in discussion with UK and Nepali pediatricians and clinical psychologists in line with the gold standard WHO guidance on adaptation of English language tools (World Health Organization 2013). All of the team involved in the translation were bilingual Nepali/English except for one (UK pediatrician). In Nepal, we discussed the translation with a small group of European and Nepali researchers, pediatricians and clinical psychologists. Team members’ experience of autism varied widely from those for which a major part of that person’s role related to diagnosis and management of autistic children and young people (e.g., Nepali clinical psychologist, pediatrician) to those who had little to no experience of working with autism (Nepali researchers). We reviewed the cultural equivalence of each item and underlying concepts in light of the Nepalese constructions of childhood, maturity and the ethnopsychological concepts of the mind (Kohrt and Harper 2008; Medeiros 2014). This allowed us to elicit the most appropriate experiential equivalent in the Nepali language.

#### Acceptability Study

The final, qualitative arm of the adaptation study was conducted in both rural (Makwanpur) and urban (Kathmandu) Nepal in the spring of 2014 as part of a larger qualitative study exploring Nepali parents and professionals’ understanding of typical and atypical child development (Heys et al. 2016). The translated version was tested for acceptability in three focus groups of parents (n = 28; 10 Kathmandu-based mothers, fathers and/or carers with a child with a known diagnosis of autism and 18 rural mothers, fathers and carers from lower caste and ethnic groups [*janajatis*] with a child(ren) without a known diagnosis of autism).

The main outcome of interest for this qualitative arm was the acceptability of the AQ-10 for use as a screening tool. The Kathmandu-based participants were purposefully recruited by our collaborators Autism Care Nepal Society (ACSN) with the inclusion criteria being: parent and/or main carer of a child (aged up to 18 years) with a known diagnosis of autism (confirmed by the clinical team at ACSN from their records). There were no exclusion criteria. These parents were mostly from a high caste, middle class and educated background living in the capital, Kathmandu. The Nepali caste system is a method of social stratification practiced by most but not all ethnic groups in Nepal.

The Makwanpur-based parents were recruited in collaboration with our partners Mother and Infant Research Activities (MIRA). The inclusion criteria were parent/carer and/or grandparent of a typical child of any age. The exclusion criterion was any known diagnosis of atypical child development (including autism). Participant characteristics for these focus groups are shown in Table 1.

**Table 1:** Participant characteristics

Participants	No. of focus groups	N (total)	Rural/urban	Gender ratio (F:M)	Participant age range (years)	Children’s age range (years)	Ethnicity	Education level	Occupation (own)	Occupation (spouse)
Parents of children without a known diagnosis of autism	2	9	Rural	9:0	20–50	< 1–31	3, 8	NR	NR	NR
		9	Rural	2:1	20–60	3–31	1, 2, 6, 7	NR	NR	NR
Parents of at least one child with a known diagnosis of autism	1	10	Urban	7:1^a^	27–43^a^	3–14 ^a^	NR	Uni Grad (7); NR (2)	Prof (7); NR (2)	prof (7), NR (2)

*F* female, *M*: Male, *N* number of participants, *FGD* focus group discussion, *NR* not recorded, *Prof* professional occupation, *Uni grad* university graduate;

^a^Data for participant age and gender and participants’ children’s ages, and education level and occupation are missing from 2 participants; Ethnicity: 1 = Brahman, 2 = Chhetri, 3 = Newar, 4 = Kami, 5 = Damai, 6 = Tamang. 1, 2 are high caste people and are highly privileged. Three are not high caste but economically privileged people, 4 and 5 are low caste people, so called “untouchables” and economically poor 6 are hill ethnic people and do not belong to the caste system. Own occupation included: non-manual working, nursing and OT assistant, assistant supervisor city bank, nursing and autistic parent’s child trainer. Spouse occupation included: doctor, businessman, operational risk manager in bank and engineer

The same research team adapted the content and the approach used in the focus groups to ensure cultural validity. We also ensured that the content discussed was accessible for the participants who had lower literacy levels, by simplifying the language, and limiting reference to abstract concepts. The approach used in the delivery of the focus group was also tailored to address the power imbalance between some of the participants (rural uneducated women) and the representations of the researchers (high caste, some male, educated middle class from the capital, Kathmandu)—by ensuring the lead facilitators were female and of a lower caste. A fourth additional workshop was held with parents of autistic children to help clarify any uncertainties around Nepali language terms for atypical child behaviors as autistic symptomatology is common among children with atypical child development.

In the initial focus groups, each of the ten questions in the AQ-10 were considered in turn, participants were asked to discuss each statement/question and consider whether each statement/question described usual or unusual child behaviors. We used the translated version of the tool with both groups of parents for consistency as this was the version we were intending to use in the prevalence study. This procedure was essential for understanding whether the final translated version (not previous untranslated/original versions) was acceptable. All participants were asked how they felt about discussing the questions and whether other parents (of children with or without a known diagnosis of autism) would be willing and able to answer the questions about their child. This discussion was facilitated using prompts such as “Do you think these questions are easy or difficult to answer?” and “Do you think parents will mind answering these questions about their children?”. Each item was discussed until there was consensus from focus group participants that the items would be acceptable for completion by Nepali parents. All participants consented for the focus group discussions to be recorded.

#### Analysis of Qualitative Data

Interview recordings were transcribed verbatim and subjected to thematic analysis following Braun and Clarke (2006) through NVivo10 (QSR International Pty Ltd 2012) as well as manually by three of the authors. The analytic process was iterative and inductive in nature. The authors independently familiarized themselves with the data and met regularly to discuss preliminary themes and codes, resolve discrepancies and decide on the final themes and subthemes.

### Prevalence Study

#### Prevalence Study Design and Timeframe

The prevalence study was a cross-sectional study of 4098 children from Makwanpur district, rural Nepal conducted between Jan 2014 and Jan 2015 in two waves of interviews and data collection. Each wave of data collection item was preceded by a pilot of data collection procedures. These 4098 children were recruited within the broader context of a long-term follow-up study (Heys et al. 2016) of a cluster randomized controlled trial (RCTcRCT) of participatory women’s groups in rural Nepal to improve neonatal survival (2001–2003) (Manandhar et al. 2004). The follow-up study was designed to examine the long-term impact on disability-free survival in mothers and their offspring enrolled in the original trial. Participants were recruited from Makwanpur district only. The inclusion criteria were all children whose mothers had been pregnant during the original trial period (2001–2003) and who were alive and willing to participate in the follow-up study.

Full details of the original trial can be found elsewhere (Manandhar et al. 2004). In brief, the initial trial intervention consisted of community-based perinatal participatory women’s groups facilitated by local women (non-health professionals). The groups investigated health issues around pregnancy, childbirth and newborn health through monthly meetings over 3 years. The primary outcome was neonatal mortality rate. Secondary outcomes included maternal mortality at 4 weeks. The study demonstrated a 30% reduction in neonatal mortality and a 78% reduction in maternal mortality in intervention clusters, compared with controls. (Manandhar et al. 2004). Pregnancies were followed until 4 weeks after delivery. (Manandhar et al. 2004; Osrin et al. 2003). The study was registered as an International Standard RCT (ISRCTN31137309).

#### Prevalence Study Procedures

In 2014–2015, two rounds of household interviews were conducted 6 months apart on all surviving and willing participants of the original trial (Haworth et al. 2017; Heys et al. 2016). Children from all clusters were included.

In the first wave of data collection, the questionnaire administered assessed survival, age and working memory for both mothers (10-word recall) and children (digit recall forward and backward). Mothers were also asked about reproductive history, literacy, smoking, diagnosis of hypertension and diabetes and psychological wellbeing. Additional child-specific topics included questions about childhood development and disability, school attendance and performance. Topics relating to the family overall included socio-economic status (as measured by household asset, land, house and animal ownership) and household occupation. Blood pressure and anthropometric measures were taken from both mothers and children.

In the second wave of data collection 6 months later, child-specific topics included the Nepali AQ-10 (written version, described herein; see Appendix Table 5) and child pubertal status (Petersen et al. 1988); maternal-specific questions included an assessment of maternal empowerment (Cunningham et al. 2015) and perceived social support (Kvaal et al. 2014); and family-specific questions focused on an assessment of family socio-economic status using the Oxford Multi-poverty Index (Alkire 2010). Critically, data pertaining to this autism prevalence study were collected in round 2 of the follow-up study only.

#### Prevalence Study Sample Size

**Fig. 1:**
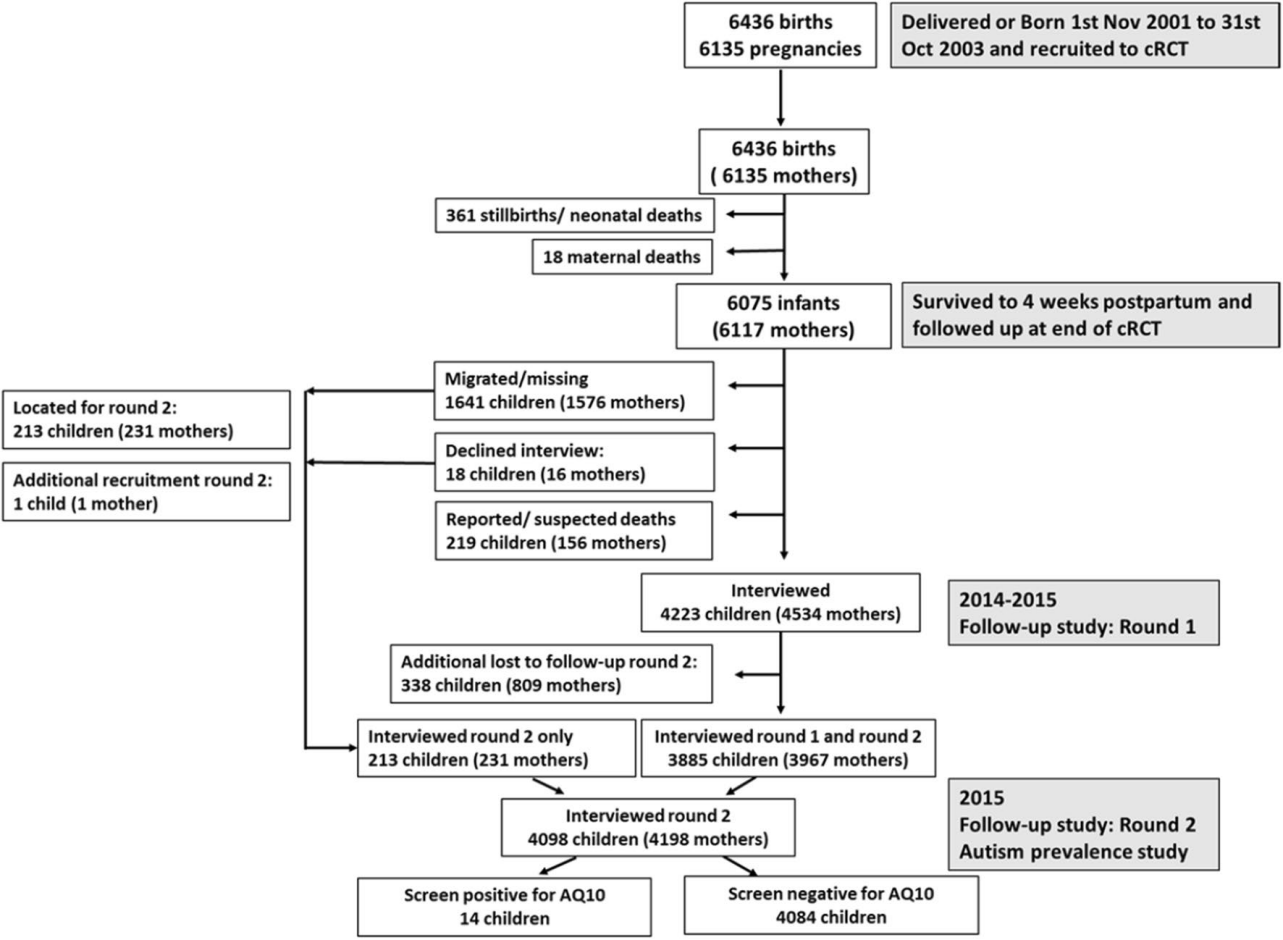
Participant flow chart from baseline to follow-up in rounds 1 and 2

In total, we conducted face-to-face interviews with 4222 (wave 1) and 4098 (wave 2) maternal-infant dyads. Figure 1 shows the flow of participants from the original trial population through to the AQ-10 follow-up in wave 2.

#### Prevalence Study Pilot Studies

Pilot studies were carried out for both waves of data collection. Research tools for the first round were piloted with 531 mother–child pairs, chosen randomly from original records in six clusters (three control and three intervention). The six clusters were randomly selected stratified by original trial allocation status. The first 100 mother–child pairs were then selected by birth date from the six clusters for the pilot study, with an aim to assess approximately 500 mother–child pairs. Of the 531 mother–child pairs who participated in wave 1 data collection, 152 of these were selected at random to participate in wave 2, 6 months later. Analysis of pilot data and field interviewer feedback were used after both pilots to revise the final questionnaires and databases and to provide additional field worker training (e.g., around measurement of body size and blood pressure). Following the pilot studies, minor edits were made only to other parts of the questionnaire and none to the disability questions (round 1) and Nepali AQ-10 (round 2). Therefore, results from the pilot and main study are presented together for the purposes of these analyses.

#### Prevalence Study Outcome Measures

Two screening tools were used for the primary and secondary outcomes, namely the adapted Nepali version of the AQ-10 and the Module on Child Functioning and Disability (MCFD), respectively. The primary outcome of interest was likelihood of autism measured by the Nepali AQ-10 and defined as a score of 6 or more out of 10 (cf. Allison et al. 2012).

The secondary outcome of interest, collected during wave 1 of data collection, was report of social and communication difficulties, as well as physical, learning and behavioral disability using the Module on Child Functioning and Disability (MCFD) produced by UNICEF and the Washington Group on disability statistics (UNICEF 2014) for use in children and young people aged 2–17 years. The MCFD is a new screening tool which builds on the established short set of questions for adult disability screening (Washington Group on Disability Statistics 2006; Washington Group on Disability Statistics and; UNICEF 2014), and is being developed as a gold standard epidemiological measure of disability prevalence. The screening tool is in the final phases of validation testing and we received permission to use it in the current study. It is carried out by interview with the child’s main caregiver to assess functioning across seven core functional domains, with a total of 19 questions, namely: speech and language, hearing, vision, cognition, mobility, self-care and emotions and behavior (see Appendix Table 6). With the exception of three questions, participants rate each item on a scale ranging from *no difficulty* to *cannot do at all*. The three exceptions are questions around the use of hearing aids/glasses (yes/no response) and the question around propensity to feelings of sadness (three possible responses: *the same or less, more* or *a lot more* than age matched comparisons). Owing to the current absence of validity data on the questions around emotions and behaviors the UNICEF and the Washington Group on disability statistics have advised the definition of disability to be the report of *at least some difficulty* in at least one of speech and language, hearing, vision, learning, mobility and motor skills—the core functional domains, termed here as MCFD-core (Washington Group on Disability Statistics). Additional to questions on these seven domains, questions on behavior, attention, relationships, playing and coping with change were used as a comparative with the AQ-10 interview—termed here as MCFD-extended. A positive screen for these questions was defined as reporting *at least some difficulty* in each area (Washington Group on Disability Statistics). Sensitivity analysis was conducted using more stringent definition of a positive screen defined as *a lot of difficulty* or *cannot do at all* in at least one of these domains (as is typically used in the adult Washington disability screening tool).

#### Supplementary Questions

In wave 1 of screening, following the administration of the MCDF, parents were asked a series of questions around what support they received for their child, if they had screened positive for disability. In particular, they were asked if they had applied for, had received and/or had used a government disability card. These cards have four color coded categories: red (completely affected); blue (severely affected); yellow (moderately affected); white (persons with mild or ordinary disabilities). Persons who have these cards have additional financial benefits from the government and also priority over some government transport and health care. Participants were also asked about school attendance (historical and current) and whether they had received any support (in the form of counselling, medical equipment, transport or financial support) from non-government organizations (NGOs). Weight and height measured in wave 1 were used to estimate stunting of growth.

#### Statistical Analysis

Quantitative statistical analyses were performed using Stata version 14.0 (StataCorp., 2015). Fischer’s exact test and Chi-squared tests were used as appropriate for univariate analysis of categorical variables and t-tests for univariate analysis of continuous variables. A p-value of less than or equal to 0.05 was interpreted as being statistically significant.

Ethical approval for the follow-up study was obtained both from Nepal Health Research Council and the University Research Ethics Committee. All participants gave informed *verbal* consent prior to participation, which is more culturally appropriate in this setting than written consent and suited to the low educational achievement/level and literacy rates of the participants.

## Results

### Preliminary Screening-Tool Acceptability Study

Parents of children with and without a known diagnosis of autism considered the AQ-10 items to be acceptable and straightforward to answer (see Table 2)—but not without caution. We identified two themes in parents’ responses reflecting the potential challenges related to probing for atypical behaviors and/or autism. They suggested that parents of children with no known diagnosis of autism (1) would not know enough about child development to be able to recognize atypical behaviors and (2) would find a potential diagnosis of autism challenging to accept or endorse. One parent of an autistic child summed up this latter point: “They will not take it [i.e., a diagnosis of autism] easy. Nowadays, all the parents expect that my son will be a big businessman, engineer and doctor. But they don’t expect anything from the autistic children but it does not mean that autistic children have no capacity. On average, they think autistic children can’t do anything and they feel them as a useless thing of society. If we try to make them aware, they will not hear us. They think spending money on autistic children is like putting water on sand.”

**Table 2:** Sample quotes from focus groups exploring the perceived acceptability of the AQ-10 by the two parent groups (those with and those without a child with a known diagnosis of autism) depending on whether the parent answering the AQ-10 had a child with or without a known diagnosis of autism

	Perceived acceptability if parents answering the AQ-10 had a child without a known diagnosis of autism	Perceived acceptability If parents answering the AQ-10 had a child with a known diagnosis of autism
Feedback from parent group		
Parents of children without a known diagnosis of autism	“No, they will not mind”	**“**They mind if their children suffer”
	“It is easy if we know how to speak”	“Perhaps, it is easy”
	“It is easy that we answer as we thought”	“Perhaps, they feel difficult because their children are suffering from that type of problems”
	“This is all about domestic question which we have already done in our daily lives so we feel it is easy”	
Parents of children with a known diagnosis of autism	“They can’t” [answer the questions] “most of the parents can’t give answers who don’t know about autism”	“It is usually happening”
	“Firstly, they will not [be] willing to give answers and secondly they will not accept to talk with you saying it is useless”	“[because] we already know about that and we are facing that too”
	“They can’t say as we did”	“We didn’t feel it difficult, it is normal”
	“Normal children’s parents also will not give answer. They don’t in how much communication and social behaves develops in children according to their age grow up. They don’t know because of lack of awareness”	
	“Most of the parents can’t give answers who don’t know about autism. Firstly, they will not [be] willing to give answer and secondly they will not accept to talk with you saying it is useless. They can’t say as we did”	

### Prevalence Study

Of the 4098 parents of surviving children interviewed in round 2 for this autism prevalence study, 14 scored 6 or greater out of 10, indicative of autistic symptomatology. Mean AQ-10 scores for those who screened positive on the AQ-10 was 7.9 (SD 1.5), with median of 8 (interquartile range 7–9); for those screening negative, 0.1 (SD 0.5), median 0, 95.4% of the cohort scored 0 out of 10. In these 4098 children, the prevalence of disability using the most inclusive definition of disability as *at least some difficulty* in one or more of the core domains was 7.4%. Prevalence of disability using more stringent cut offs provided the following prevalence: MCFD-core reporting *a lot of difficulty* in at least one domain: 1.0% and MCFD-core reporting *cannot do at all* in at least one domain: 0.3%.

**Fig. 2:**
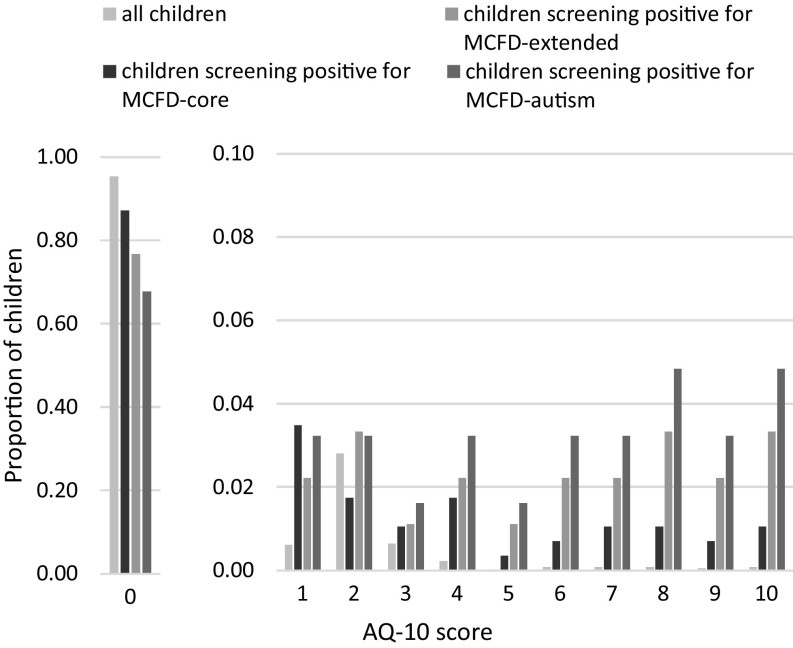
Proportion of children by AQ10 score. Proportions are shown for four groups of children, namely: (1) all children, (2) children who screen positive for MCFD-core; (3) children who screen positive for MCFD-extended and (4) children who screen positive for MCFD-extended questions most intuitively associated with autism symptomatology—MCFD-autism. *AQ-10* Autism quotient-10, *MCFD* module of child functioning and disability

Figure 2 shows the proportion of children by AQ-10 score from 0 to 10 and the proportion of children by AQ-10 score from 1 to 10 inclusive, thus more clearly visualising the distribution in scores of 1 and above. These scores are shown for four groups, namely: (1) all children, (2) children screening positive for the MCFD-core disability questions, (3) children screening positive for the MCFD-extended questions and (4) children screening positive for the 3 questions most intuitively associated with autistic symptomology—denoted in Fig. 2 as MCFD-autism. The AQ-10 scores for children who scored postitive for the extended MCFD questions and for the MCFD-autism questions were spread more evenly across the potential AQ-10 scores from 0 to 10, with a greater number of children showing higher AQ-10 scores. Mean AQ-10 scores for the four groups of children were as follows: (1) all children: M = 0.12 (SD = 0.66); (2) MCFD-core disability: M = 0.55 (SD = 1.82) and (3) MCFD-extended: M = 1.36 (SD = 2.88) and (4) MCFD-autism: M = 1.94 (SD = 3.31).

Table 3 describes key characteristics of the 14 children who scored 6 or more out of 10 in the AQ-10. Almost two-thirds (65%) were male, which compares to 51% males in the cohort as a whole, and their mean age, 11.4 years (range 10.5–12.3), was similar to the entire cohort. Thirteen out of 14 screened positive for disability on the MCFD and 12 screened positive for difficulties in communication, forming relationships with others, coping with change, playing with other children and attention (see Table 4). None of the children had a known diagnosis of autism. One child screened positive for AQ-10 (score 6 out of 10) but negative for all of the disability screen (Table 3: Child 1). Out of the 14 children screening positive, this child was one of only 4 children who attended school and the only child of the 14 who was able to complete the digit recall forward and backward scoring below average on both scores (digit recall forward 4: M for cohort = 4.8, SD = 1.1; digit recall backward 3: M = 3.1, SD = 1.6). The majority of the 14 children screening positive (71%) did not attend school, compared with 4% of the cohort, and 83% had stunted growth compared with 39.6% of the cohort. A child is said to have stunted growth when their height for age is < 2 standard deviations of the WHO child growth standards median. Five families had applied for the government disability card (see Table 3) and, of the four who had received one, two had used it. The same two families had received financial support from an NGO. Of note, children who screened positive for the AQ-10 were equally as likely to be in the intervention or control arm of the original trial with 7 children in each trial arm (p-value for chi-squared, 0.945).

**Table 3:** Characteristics of children with scores of 6 or more out of 10 on the AQ-10

Child	Gender	Age (years)	Maternal age at follow-up (years)	Family ethnicity^a^	AQ-10 score	Positive or negative screen for disability	MCFD disability score (out of 12)	Ever attended school	Attends school now
1	Girl	12.4	38.1	Tamang	6	Negative	–	Yes	Yes
2	Boy	12.3	37.3	Brahmin	8	Positive	7	No	No
3	Boy	11.7	38.8	Tamang	7	Positive	11	No	No
4	Girl	10.7	29.7	Pariyar	8	Positive	11	Yes	Yes
5	Boy	10.8	48.3	Tamang	10	Positive	9	No	No
6	Boy	11.0	40.5	Tamang	10	Positive	8	No	No
7	Boy	11.6	35.4	Brahmin	9	Positive	8	No	No
8	Boy	12.1	55.8	Tamang	8	Positive	12	Yes	Yes
9	Girl	10.5	36.2	Chhetri	9	Positive	10	No	No
10	Girl	11.0	42.3	Tamang	6	Positive	9	No	No
11	Girl	12.3	32.4	Tamang	7	Positive	11	No	No
12	Boy	11.5	39.0	Tamang	6	Positive	9	No	No
13	Girl	11.7	39.3	Pariyar	10	Positive	10	No	No
14	Boy	11.9	40	Tamang	7	Positive	2	Yes	Yes

Child	Stunted growth	7 Digit recall (forward) (mean 4.8, SD 1.1)	7 Digit recall (back; mean 3.1,SD 1.6)	Applied for disability card	Has disability card	Colour of disability card^b^	Used disability card?	Received support from NGO	What type of support from NGO
1	No	4	3	–	–	–	–	–	–
2	No	0	0	Yes	Yes	Blue	No	No	–
3	Stunted	0	0	Yes	Yes	Blue	Yes	Yes	Financial
4	Stunted	3	0	No	–	–	–	No	–
5	Stunted	0	0	No	–	–	–	No	–
6	Stunted	0	0	No	–	–	–	No	–
7	–	0	0	No	–	–	–	No	–
8	Stunted	0	0	Yes	Yes	Blue	No	No	–
9	Stunted	0	0	No	–	–	–	No	–
10	Stunted	0	0	No	–	–	–	No	–
11	Stunted	0	0	Yes	Yes	Red	No	No	–
12	Stunted	0	0	Yes	No	–	–	No	–
13	–	0	0	Yes	Yes	Red	Yes	Yes	Financial
14	Stunted	3	0	No	–	–	–	No	–

*MCFD* Module on childhood functioning and disability, *NGO* non-government organisation

^a^Ethnicity: Brahman and Chhetri are high Caste people and are highly privileged. Tamang are hill ethnic people and do not belong to the caste system, Pariyar are are low caste people, so called “untouchables” and economically poor

^b^Government disability card: red: completely affected; blue: severely affected; yellow: moderately affected; white: persons with mild or ordinary disabilities

**Table 4:** Numbers of children screening positive for both the AQ-10 and overall disability and specific questions around social and communication

Disability measures by MCFD		AQ-10		p-value (Fischer’s exact)
		Positive	Negative	
Overall disability				
Disability (MCFD-core)^a^	Positive	13	274	< 0.001
	Negative	1	3596	
All disability (MCFD-core plus extended)^b^	Positive	13	213	< 0.001
	Negative	1	3558	
Subtype disability				
Physical disability	Positive	12	222	< 0.001
	Negative	2	3648	
Learning disability	Positive	13	89	< 0.001
	Negative	1	3782	
Behavioural disability	Positive	11	53	< 0.001
	Negative	3	3832	
Difficulties in coping with change	Positive	12	34	< 0.001
	Negative	2	3837	
Difficulties in social relationships	Positive	12	17	< 0.001
	Negative	2	3854	
Difficulties in playing with others	Positive	12	25	< 0.001
	Negative	2	3846	
Attention difficulties	Positive	12	32	< 0.001
	Negative	2	3839	

*MCFD* Module on childhood functioning and disability

^a^Measured by positive report of difficulty in domains of seeing, hearing, speech and language, mobility, self-care and cognition

^b^Including on six core domains and the additional report of emotional and/or behavioural difficulties

Using questions from the MCFD extended screen, we created a composite score by summing the three questions most intuitively associated with autism symptomatology, namely coping with changes, social relationships and playing with others—denoted in Fig. 2 as MCFD-autism. Figure 2 shows a histogram of this composite score from the subset disability screening, excluding those children with a score of zero. Of the 4222 children, this composite score identified 66 (1.6%; 39 males; M age = 11.4 years, SD = 0.6) who screened positive for reporting a problem in one or more of these areas. Of these 66 children, 62 (94%) received the AQ-10 in round 2, 6 months later, with mean AQ-10 scores of 1.94 (SD = 3.31, range 0–10) – which were significantly higher (p < 0.001) than the scores from the remaining cohort (n = 3,822; M = 0.1, SD = 0.47). Of these 62 children, 20 (32%) scored 1 or more out of 10 on the AQ-10.

## Discussion

This is one of only two published studies to date to have estimated the prevalence of autism in a rural LIC setting. The study showed that the adapted AQ-10 was acceptable to groups of parents of children both with and without a known diagnosis of autism. Of the 4098 children sampled, 14 scored positive for autistic symptomatology. The demographic, anthropometric, clinical and educational characteristics and gender ratio of these 14 children indicate that the AQ-10 administered with a threshold score of 6 or above in this population is likely to identify children with complex needs and more likely more severe autism. 66 out of 422 children screened positive for the three questions most intuitively associated with autism symptomatology from the MCFD disability screening tool. A comparison of scores from the AQ-10 to those from the MCFD administered 6 months earlier provided some evidence of the clinical validity of the AQ-10. Likewise, the comparison of scores from these two tools provided evidence of the potential of MCFD, at least the section pertaining to child behavior, to identify successfully children within the population with atypical child development and behaviors.

There is no population-based study with which to compare these AQ-10 scores. The original paper describing the AQ-10 as a short screening tool was tested on UK cases (autistic children and adults) and controls (children and adults without a known diagnosis of autism) (Allison et al. 2012). The autistic adolescents scored a mean of 8.40 (SD 1.69) and adolescent controls a mean of 1.78 (SD 1.80). The AQ-10 scores for UK cases were therefore not dissimilar to those from this cohort who screened positive with AQ-10 scores (M = 7.9; SD 1.5). However, the Nepali population mean scores were much lower than those of the control group tested in the UK study.

If the AQ-10 screening tool is as sensitive and specific in the Nepali population as it is in the UK, the current results would give an estimated true prevalence of 3 in 1000 (95% confidence interval: 2–5 in 1000) (Brown et al. 2001). The current population of children under 18 years in Nepal is a little over 11.6 million. If confirmed, these prevalence estimates would equate to 34,803 children and young people currently living in Nepal (range 23,203–58,007) with a potential diagnosis of autism. The number of children with a current diagnosis of autism (n = 107); Kathmandu Valley, 2012 estimates (Autism Care Society Nepal) is substantially lower than this figure. This estimated prevalence of 3 per 1000 is lower than that in HICs, which is reported as 10–20 per 1000 (Elsabbagh et al. 2012), and lower than the only other LIC study showing Ugandan population estimates in 1–10-year-olds to be 12–13 per 1000 (Kakooza-Mwesige et al. 2014). Nevertheless, this estimate is higher than global median estimates (1.7/1000) and estimates from a recent study in rural India 0.9 per 1000 for all children in the full cohort of 11,000 children aged between 1 and 10 years and 1.1 per 1000 in the rural cohort (Raina et al. 2017). In the latter study, urban rural abode and higher socio-economic class were associated with reduced prevalence. The closest areas geographically, other than India, in which there is some (but still not a significant amount of) research on autism prevalence available are Sri Lanka and Indonesia. One, relatively old Indonesian-based study estimated 1.7 cases of autism per 1000 of the population (Wignyosumarto et al. 1992). In comparison, a study based in Sri Lanka placed the estimate as high as 10 per 1000 (Perera et al. 2009).

Of those children who screened positive for autism symptomatology, almost all also screened positive for physical, learning and behavioral disabilities. Given these two screening tools were delivered approximately 6 months apart, this finding provides preliminary evidence that our modified AQ-10 is a valid measure of atypical child behavior. It also suggests that those children who screened positive for autism symptomatology on the AQ-10 have evidence of multiple impairments and so most likely represent the severe end of the spectrum of potential clinical presentations of autism. This is also reflected in the gender ratio (1.4:1, M:F), showing a much higher number of females than is typically reported in the full spectrum of autism, around 4:1 (Fombonne 2009); though see (Loomes et al. 2017). In autistic children who are cognitively less able, the gender ratio falls from 10:1 to be closer to 1:1 (Volkmar et al. 1993). It is therefore likely that our estimated prevalence of 3 per 1000 is an *under*estimation of the true prevalence of autism within this population. This is also reflected in the number of children (66 of 4222) screening positive for difficulty in at least one of the areas of coping with changes, social relationships and playing with others. These questions have not yet been validated or indeed tested in any way as a screen for autism. Yet if it were assumed that a positive screen was indicative of likelihood of autism, this figure would equate to an estimated prevalence of 16 per 1000, much more similar to HIC estimates and those from Uganda. Indeed, the Ugandan study used an adaptation of the Ten questions questionnaire that was the basis for the MCFD used in this study (Kakooza-Mwesige et al. 2014).

In addition to being more likely to screen positive for physical, learning and behavioral disabilities, children who screened positive for autism symptomatology were more likely to be stunted and to have cognitive difficulties (as measured by marked difficulties completing the forward and backward digit recall, a measure of working memory). The majority of these children were not attending education and were unlikely to be receiving any financial support despite having significant difficulties. An 83% stunting rate is substantial. Nutritional deficits in children with disabilities and learning difficulties are common and can not only be a cause of cognitive deficits, but also contribute to the failure to reach full developmental potential in the presence of a developmental condition. These limited, but important descriptive data support the general opinion that children with all kinds of atypical child development and disability are a highly vulnerable, disadvantaged group, especially in resource-limited settings such as Nepal (UNICEF 2013).

The strengths of our study include (1) its novelty—to our knowledge there is only one other published study of prevalence estimates in a LIC; (2) the interview of families within households, thus including children who were not present in school; (3) our restricted age range, thus rendering age range less of a confounding variable; and (4) our cultural adaptation of the screening tool using qualitative methods in collaboration with local stakeholders (Stewart and Lee 2017). Also, to our knowledge, no other study has incorporated cultural constructions of mind/emotions and local notions of childhood/children in the translation and piloting of the AQ-10 or similar tools. We also situated our prevalence estimate within the context of other important data, including disability screen, school attendance and growth with which to explore characteristics of those screening positive for AQ-10.

There are, however, limitations to our study. First, the cohort is derived from mother-infant dyads who were enrolled in a perinatal trial. Nevertheless, children who screened positive for the AQ-10 were equally as likely to have been born into the villages enrolled in the intervention as those villages enrolled in the control. Second, the MCFD is under development and indeed since the commencement of this study minor edits have been made to the questions. In addition, early testing in India and Cameroon, coupled with the prevalence found here, suggest cultural interpretation of degree of difficulty may greatly influence reporting (Mactaggart et al. 2016). For instance, prevalence of reporting *at least some difficulty* in at least one domain was 35% and 64% in India and Cameroon, respectively, whereas prevalence of reporting *a lot of difficulty* or *cannot do* in at least one domain was 4 and 9% again in India and Cameroon, respectively (Mactaggart et al. 2016). Thus, our comparison of MCFD-extended questions with AQ-10 here should be considered exploratory. Notably, however, our prevalence estimate of 7.4% is in keeping with a systematic review of the global estimates of childhood disability in LMICs which suggested that despite a wide range in estimates (0.5–18%), the majority clustered around 5–10% (Maulik and Darmstadt 2007).

Future research is required to validate the AQ-10 and the MCFD-core and -extended modules through in-depth comprehensive assessments of high-scoring children and a representative sample of low-scoring children. Given the wide range of perinatal data available for this cohort, we also have the unique opportunity to conduct an exploratory study around the association of perinatal factors with likelihood of autism symptomatology in a population of children with poor nutrition (40% stunting at mean age of 11.5 years). Such prevalence research, however, also raises serious ethical concerns, including the possibility of disclosure of likelihood of and/or even a diagnosis of autism in a population for whom there is no term for autism (see Heys et al. 2016) and there is very little health or education provision to support children and families with a diagnosis of autism. These issues surrounding the ethics and logistics of diagnostic disclosure were precisely the ones that our focus group participants perceived to be of concern. There are also no validated diagnostic tools available for use in the Nepali population, rendering a validation study even more challenging. Thorough examination of these issues with Nepali parents and practitioners is essential prior to pursuing further the validation study of the AQ-10, including examining the potential impact of disclosure of a diagnosis of, or likelihood of a diagnosis of autism about which very little is known in most resource-poor settings like Nepal. Finally, future research in this area should explore the potential impact of caste/ethnicity and rural/urban divide on understanding of autism and its implications.

In our qualitative study of Nepali parents’ and professionals’ understanding of typical and atypical child development (with an emphasis on autistic symptomatology), we found that parents of children without a diagnosis of autism and professionals in general had little explicit awareness of autism (Heys et al. 2016). Only parents of autistic children, pediatricians and the disability sector worker identified behaviors typically associated with autism as ‘autistic’. Other participants, including parents of children without an autism diagnosis, primary and early child development teachers, community health workers and faith healers, used distinctive terms, such as “stubborn” and “insisting” to distinguish vignettes of autistic children from vignettes of children with other developmental conditions. Most participants felt that environmental factors, including in-utero stressors and birth complications, parenting style, and home or school environment, were key causes of atypical child development and further called for greater efforts to raise awareness and build community capacity to address autism. Thus, the preliminary prevalence estimate reported herein, combined with complementary research showing the lack of awareness of autism by Nepali professionals and parents, demonstrates a substantial unmet need and stresses the importance of developing services to support families and children with atypical development in LIC settings.

### Electronic supplementary material

Below is the link to the electronic supplementary material.

Supplementary material 1 (DOCX 45 KB)

## Appendix

See Tables 5 and 6.

**Table 5:** AQ-10 questionnaire (English/Nepali)

1.यो बच्चाले जुनसुकै कृयाकलाप गर्दा सधै एकै तरिकाले गर्ने वा बुझ्ने गर्छ? S/he notices patterns in things all the time. 1= गर्छ Yes 0= गर्दैन No 2. कुनै पनि बस्तुगत अवलोकन गर्दा समग्र रुपमा नभएर सुक्ष्म रुपमा अवलोकन गर्छ? S/he usually concentrates more on the small details rather than the whole picture. 1= गर्छ Yes 0= गर्दैन No 3.यो बच्चाले सामाजिक समुहमा बिभिन्न प्रकारका व्यक्तिहरुको बार्तालापलाई सजिलै पहिल्याउन सक्छ? In a social groups/he can easily keep track of several different people’s conversations. 1= सक्छ Yes 0= सक्दैन No 4. एउटा कृयाकालापबाट अर्को कृयाकालापमा सहजै सहभागी हुन् सक्छ र फर्केर पुरानै कृयाकलाप जारी राख्न सक्छ? If there is an interruption, s/he can switch back to what s/he was doing very quickly. 1= सक्छ Yes 0= सक्दैन No 5. यो बच्चाले आफ्ना सहकर्मीहरुसंग कसरी कुराकानी गरिरहने भनेर जान्दैन? S/he does not know how to keep a conversation going with his/her peers
1= जान्दछ Yes 0= जान्दैन No 6. यो बच्चा सामाजिक गफगाफ (कुराकानी) गर्नमा राम्रो छ ? S/he is good at social chit-chat
1= छ Yes 0= छैन No 7. यो बच्चा सानो छंदा ऊ साथीहरुसंग काल्पनिक खेल खेल्न मन पराउँथ्यो ? When s/he was young s/he used to enjoy playing games involving pretending with other children?
1= पराउँथ्यो Yes 0= पराउँदैनथ्यो No 8. कथा पढ्दा वा सुन्दा कथाको पात्रको भावना तथा मनसाय अड्कल लगाउन गाह्रो हुन्छ ?, s/he finds it difficult to imagine what it would be like to be someone else 1= हुन्छ Yes 0= हुँदैन No 9. यो बच्चाले सामाजिक अवस्था सजिलो महसुस गर्छ/ S/he finds social situation easy 1= गर्छ Yes 0= गर्दैन No 10. यो बच्चालाई नयाँ साथि बनाउन गाह्रो हुन्छ/ S/he finds it hard to make new friends 1= हुन्छ Yes 0= हुँदैन No

**Table 6:** Module on child functioning and disability

Seeing	Children aged 2–17	Does [name] wear glasses or contact lenses?	1 = Yes 0 = No
		[*if wears glasses*] Does [name] have difficulty seeing, when wearing his/her glasses? Would you say…	1 = No difficulty 2 = Some difficulty 3 = A lot of difficulty 4 = Cannot do at all
		[*If child does NOT wear glasses*] Does [name] have difficulty seeing?	1 = No difficulty 2 = Some difficulty 3 = A lot of difficulty 4 = Cannot do at all
Hearing	Children aged 2–17	Does [name] use a hearing aid?	1 = Yes 0 = No
		[*If child uses a hearing aid*] Does [name] have difficulty hearing, when using his/her hearing aid(s)?	1 = No difficulty 2 = Some difficulty 3 = A lot of difficulty 4 = Cannot do at all
		[*If child does NOT use a hearing aid*] Does [name] have difficulty hearing?	1 = No difficulty 2 = Some difficulty 3 = A lot of difficulty 4 = Cannot do at all
Walking	Children aged 5–17	Compared with children of the same age, does [name] have difficulty walking 500 yards/meters on level ground? That would be about the length of 5 football fields	1 = No difficulty 2 = Some difficulty 3 = A lot of difficulty 4 = Cannot do at all
		Compared with children of the same age, does [name] have difficulty walking 100 yards/meters on level ground? That would be about the length of 1 football field	1 = No difficulty 2 = Some difficulty 3 = A lot of difficulty 4 = Cannot do at all
Self-care	Children aged 5–17	Compared with children of the same age, does [name] have difficulty with self-care such as feeding or dressing him/herself?	1 = No difficulty 2 = Some difficulty 3 = A lot of difficulty 4 = Cannot do at all
Communication and comprehension	Children aged 5–17	Compared with children of the same age and using [his/her] usual language, does [name] have difficulty understanding other people?	1 = No difficulty 2 = Some difficulty 3 = A lot of difficulty 4 = Cannot do at all
		Compared with children of the same age and using [his/her] usual language, does[name] have difficulty being understood by other people?	1 = No difficulty 2 = Some difficulty 3 = A lot of difficulty 4 = Cannot do at all
Learning	Children aged 3–17 years	Compared with children of the same age, does [name] have difficulty learning to do new things?	1 = No difficulty 2 = Some difficulty 3 = A lot of difficulty 4 = Cannot do at all
	Children aged 5–17	Compared with children of the same age, does [name] have difficulty remembering things that they have learned?	1 = No difficulty 2 = Some difficulty 3 = A lot of difficulty 4 = Cannot do at all
Emotions	Children aged 5–17	Compared with children of the same age, how much does [he /she] worry or feel sad? Would you say… [*Read response categories*]	1 = The same or less 2 = More 3 = A lot more
Behaviour	Children aged 5–17	Compared with children of the same age, how much difficulty does [name] have controlling[his/her] behaviour?	1 = No difficulty 2 = Some difficulty 3 = A lot of difficulty 4 = Cannot do at all
Attention	Children aged 5–17	Compared with children of the same age, does [name] have difficulty completing a task?	1 = No difficulty 2 = Some difficulty 3 = A lot of difficulty 4 = Cannot do at all
Coping with change	Children aged 5–17	Compared with children of the same age, does [name] have difficulty accepting change to plans or routine?	1 = No difficulty 2 = Some difficulty 3 = A lot of difficulty 4 = Cannot do at all
Relationships	Children aged 5–17	Does [name] have difficulty getting along with children of [his/her] age?	1 = No difficulty 2 = Some difficulty 3 = A lot of difficulty 4 = Cannot do at all
Playing	Children aged 2–12 years	Compared with children of the same age, does [name] have difficulty playing with other children?	1 = No difficulty 2 = Some difficulty 3 = A lot of difficulty 4 = Cannot do at all

## Footnotes

1 Identity-first language (i.e. autistic person), opposed to person-first language (i.e., person with autism), is preferred by many autistic people and their allies (see Kenny et al. 2016) and (Sinclair 1999). Therefore, in this article, the authors use predominantly identity-first language.
