# Supplementary material for: The Estimated Prevalence of Autism in School-Aged Children Living in Rural Nepal Using a Population-Based Screening Tool
Source: J Autism Dev Disord. 2018 May 31;48(10):3483–98. doi: 10.1007/s10803-018-3610-1 (PMC6153945; doi:10.1007/s10803-018-3610-1)
Supplement: Supplementary file 1 — Supplementary material 1 (DOCX 45 KB) [file 10803_2018_3610_MOESM1_ESM.docx]

Supplementary Table 1: *Screening tools for ASD*

| Tool | Description | Length | Reported by | Age range | Sensitivity | Specificity | Target population | Free/not free | | Disadvantages | |
| --- | --- | --- | --- | --- | --- | --- | --- | --- | --- | --- | --- |
| *Tools that can be applied to older children* | | | | | | | | | | | |
| Autism Quotient (AQ)-10^1,2^ | questionnaire  (shorter version of AQ-50) | 10 item | Parent (child/ adolescent);  self-report (adult) | (1) adult,  (2) adolescent (3) child | 93%^3^ | 97%^2^ | Population based | Free^4^ | |  | |
| Social Communication Questionnaire^5^ | questionnaire | 40 item | parent | (1) < 6 years  (2) ≥ 6 years | 85%^6^ | 75%^2^ | Those screening positive in primary screen | Not free | | Primarily second-stage screen | |
| Social Responsiveness Scale (SRS)^7^ | questionnaire | 65 items | Parent and teacher | 4-18 year olds | 67%^7^ | 78%^7^ |  | For use in educational and clinical settings | | Needs teacher input | |
| Autism Spectrum Screening Questionnaire^8^ | questionnaire | 27 item | Parent/ teacher | 7 to 16 years | 91%^8,9^ | 86%^8,9^ | ‘High functioning’ ASD/  Asperger’s | Free | | Designed for screening for autistic children and young people with IQ in normal range | |
| Childhood Asperger’s Syndrome Test (CAST)^10^ | questionnaire | 37 item | Parent | 4 to 11 years | 100%^10^ | 97%^10^ | Primary school aged children (main stream) | Free | | Screening for children and young people with ‘higher functioning’ autism | |
| Developmental Behaviour Checklist–Autism Screening Algorithm^11^ | questionnaire derived from the Develop-mental Behavior Checklist for Pediatrics^11^ | 29 item | Parent | 4 to 18 years | 86%^12^ | 69%^12^ | for children with intellectual disabilities only |  | | May report false positives in children with significant behaviour problems | |
| *Tools that are only applied to younger children* | | | | | | | | | | | |
| Developmental Behaviour Checklist - Early Screen^13^ | questionnaire derived from the Developmental Behavior Checklist for Pediatrics | 17 item | Parent | 18-48 month old | 88%^13^ | 69%^13^ | Preschool children with developmental delay | |  | | Young age range; Screen for clinical population only |
| Modified Checklist for Autism in Toddlers (MCHAT)^14^ | questionnaire | 20 item with clinician follow-up for positive screen | Parent/ clinician | 16-30 month old children | 86%^15^ | 99%^15^ | Population screening tool for preschool children | | Free^16^ | | Requires parent and clinician report |
| Checklist for Autism in Toddlers (CHAT)^17^ | questionnaire and observation | 14 items focusing on joint attention and pretend play | Parent/ clinician | screening for 18-month-olds | 20-38%^17^ | 98%^17^ (as first-tier screen) | Population screening tool for preschool children | | Free | | Requires direct observation of behaviour |
| Pervasive Developmental Disorders Screening Test II^18^ | primary care screener questionnaire – Stage 1 of the full test | 22 item | Parent | 1-2 year old screening only | 92%^18^ | 91%^18^ |  | | Not free | |  |
| Screening Tool for Autism in Two year olds (STAT)^3^ | interactive observation screening | approximately 20 minutes | Clinician | 2-3 year olds only | 92%^3^ | 85%^3^ | To distinguish between ASD and other neurodevelopmental disorders | | Not free | | Mainly used as a second-stage screen  long and requires direct observation |
| Parent's Observations of Social Interactions (POSI)^19^ | questionnaire (developed from MCHAT) | 7 item | Parent | Children 16-36 months | 89%^19^ | 54%^19^ |  | | Free | |  |

1. Booth T, Murray AL, McKenzie K, Kuenssberg R, O'Donnell M, Burnett H. Brief report: an evaluation of the AQ-10 as a brief screening instrument for ASD in adults. *Journal of autism and developmental disorders* 2013; **43**(12): 2997-3000.

2. Allison C, Auyeung B, Baron-Cohen S. Toward brief "Red Flags" for autism screening: The Short Autism Spectrum Quotient and the Short Quantitative Checklist for Autism in toddlers in 1,000 cases and 3,000 controls [corrected]. *Journal of the American Academy of Child and Adolescent Psychiatry* 2012; **51**(2): 202-12 e7.

3. Stone WL, Coonrod EE, Turner LM, Pozdol SL. Psychometric properties of the STAT for early autism screening. *Journal of autism and developmental disorders* 2004; **34**(6): 691-701.

4. Centre AR. <https://www.autismresearchcentre.com/arc_tests>.

5. Rutter M., A. B, C. L. The Social Communication Questionnaire. . In: Services WP, ed. Los Angeles; 2003.

6. Berument SK, Rutter M, Lord C, Pickles A, Bailey A. Autism screening questionnaire: diagnostic validity. *The British journal of psychiatry : the journal of mental science* 1999; **175**: 444-51.

7. Constantino JN, Lavesser PD, Zhang Y, Abbacchi AM, Gray T, Todd RD. Rapid quantitative assessment of autistic social impairment by classroom teachers. *Journal of the American Academy of Child and Adolescent Psychiatry* 2007; **46**(12): 1668-76.

8. Ehlers S, Gillberg C, Wing L. A screening questionnaire for Asperger syndrome and other high-functioning autism spectrum disorders in school age children. *Journal of autism and developmental disorders* 1999; **29**(2): 129-41.

9. Posserud MB, Lundervold AJ, Gillberg C. Validation of the autism spectrum screening questionnaire in a total population sample. *Journal of autism and developmental disorders* 2009; **39**(1): 126-34.

10. Williams J, Scott F, Stott C, et al. The CAST (Childhood Asperger Syndrome Test): test accuracy. *Autism : the international journal of research and practice* 2005; **9**(1): 45-68.

11. Brereton AV, Tonge BJ, Mackinnon AJ, Einfeld SL. Screening young people for autism with the developmental behavior checklist. *Journal of the American Academy of Child and Adolescent Psychiatry* 2002; **41**(11): 1369-75.

12. Witwer AN, Lecavalier L. Autism screening tools: an evaluation of the Social Communication Questionnaire and the Developmental Behaviour Checklist-Autism Screening Algorithm. *Journal of intellectual & developmental disability* 2007; **32**(3): 179-87.

13. Gray KM, Tonge BJ. Screening for autism in infants and preschool children with developmental delay. *The Australian and New Zealand journal of psychiatry* 2005; **39**(5): 378-86.

14. Chlebowski C, Robins DL, Barton ML, Fein D. Large-scale use of the modified checklist for autism in low-risk toddlers. *Pediatrics* 2013; **131**(4): e1121-7.

15. Robins DL, Fein D, Barton ML, Green JA. The Modified Checklist for Autism in Toddlers: an initial study investigating the early detection of autism and pervasive developmental disorders. *Journal of autism and developmental disorders* 2001; **31**(2): 131-44.

16. [www.mchatscreen.com](http://www.mchatscreen.com).

17. Baird G, Charman T, Baron-Cohen S, et al. A screening instrument for autism at 18 months of age: a 6-year follow-up study. *Journal of the American Academy of Child and Adolescent Psychiatry* 2000; **39**(6): 694-702.

18. Siegel B. PDDST-II Pervasive Developmental Disorders Screening Test–II. 2004.

19. Smith NJ, Sheldrick RC, Perrin EC. An Abbreviated Screening Instrument for Autism Spectrum Disorders. *Infant Mental Health Journal* 2013; **34**(2): 149-55.
